# 5956 German affective norms for atmospheres in organizations (GANAiO)

**DOI:** 10.3758/s13428-024-02566-2

**Published:** 2024-12-18

**Authors:** Anna Eifert, Christian Julmi

**Affiliations:** 1 Department of Business Administration and Economics, FernUniversität in Hagen, Hagen, Germany; 2 FernUniversität in Hagen, Fakultät für Wirtschaftswissenschaft, Lehrstuhl für Betriebswirtschaftslehre, insb. Organisation und Planung, 58084 Hagen, Germany

**Keywords:** Affective atmospheres · Organizational atmospheres · Dictionary based language analysis · Dimensional approach · Categorical approach · Valence · Arousal

## Abstract

This article develops a comprehensive database comprising 5956 German affective norms specifically tailored for the study of organizational atmospheres through computational verbal language analysis. This dictionary adopts both dimensional and categorical approaches. The theoretical foundation of this study is the circumplex model of affective atmospheres. Similar to established methodologies, each word is rated based on the dimensions of valence and arousal. Going beyond the dimensional approach, this article introduces a classification system with 11 distinct atmospheric categories, assigning the words to their corresponding categories. This dictionary represents the first attempt to apply computer-aided text analysis (CATA) to the study of organizational atmospheres, providing a practical tool to support research in this developing area.

## Introduction

An atmosphere can be understood as “a total or partial, but in any case comprehensive, occupation of an area-less space in the sphere of that which is experienced as being present” (Schmitz, 2016, p. 4). In other words, atmospheres are pervasive, intangible phenomena that surround and influence individuals within a particular space. They are omnipresent in organizational environments, where people can often sense them immediately upon entering a room—whether it is the charged atmosphere of a meeting or the relaxed vibe of a casual conversation among colleagues. With that in mind, visualize the tense atmosphere of a meeting and then the thrilling atmosphere of celebrating an important milestone and how each makes you feel. While both situations are intense, the first one is more likely associated with negative feelings and the latter with positive ones. In contrast, the relaxed atmosphere of a Christmas party or of the dreary morning mood on a Monday are typically categorized as calm, although the two are judged quite differently. These examples show that the affective experience of environmental atmospheres can be classified according to two dimensions, which are valence (positive–negative) and arousal (calm–excited) (Russell & Pratt, 1980; Julmi, 2017a).

What is peculiar about atmospheres is that they are both object-related (as environmental qualities) and subject-related (as affective phenomena). In organizations, atmospheres significantly impact the behavior and interactions of their members (Julmi, 2017b; Jørgensen & Beyes, 2023; Julmi et al., 2024) and are increasingly the subject of empirical studies in management and organizational research (Knight et al., 2024; De Molli et al., 2020; Marsh & Śliwa, 2022; Leclair, 2023).

Employees attach significant importance to the work atmosphere; for instance, before applying to a company, they actively seek information about its overall atmosphere (Julmi et al., 2024). Hence, they look up employer reviews on platforms such as *glassdoor* and *kununu*, where employees share their experiences as well as describing and rating the work atmosphere of their (current or former) organization. As Radermacher and Herdejürgen (2022) show, the work atmosphere is decisive for recommending a company on such portals.

To research affective phenomena, computer-aided text analysis (CATA) has been established in management and organization studies (McKenny et al., 2013). Through CATA, scholars can analyze and manage large sets of qualitative data to explore phenomena of interest and improve their theory building and hypotheses testing (Short et al., 2018). Dictionary-based emotion and sentiment analysis is well established for gaining insights from unstructured data, such as social media posts or employer reviews, and converting it into meaningful information (Kour et al., 2021). For instance, CATA can reveal factors that influence individual behavior and motivation, as well as shedding light on cultural and social dynamics within organizations (McKenny et al., 2018; Short et al., 2018). In the context of atmospheres, researchers can employ such dictionaries to uncover the underlying factors that shape organizational atmospheres and understand how these atmospheres impact organizational members. The stimulus set developed in this study can thus provide a crucial foundation for theory development in this emerging area. By offering a structured framework for analyzing organizational atmospheres, it opens up opportunities to investigate key questions: How do different atmospheres affect employee well-being, productivity, or collaboration? What strategies can organizations employ to cultivate more positive atmospheres? However, despite their growing significance for organizations (Julmi, 2017c; Jørgensen & Beyes, 2023), scholars have not yet, to the best of our knowledge, used CATA to specifically analyze atmospheres in organizations.

Existing dictionaries for emotion or sentiment analysis are not suitable for studying verbal descriptions of shared atmospheres. Although emotions and atmospheres are closely related phenomena, they are distinctly different. While emotions are more subjective and private phenomena, atmospheres are a more overarching phenomenon that is attributed to the environment or a particular situation (Fuchs, 2013). Accordingly, the difference between norms for atmospheres in organizations and norms for affective words can be seen in the words themselves, which differ greatly in type and content from the words in previous dictionaries on affects. For instance, if emotions are described as sad, uneasy, angry, or happy, the corresponding atmospheres are more likely to be characterized as gloomy, eerie, heated, or exciting (Russell & Pratt, 1980). Other words such as conflict-laden, collaborative, casual, authoritarian, stuffy, inviting, sterile, dim or vibrant describe atmospheres and thus possess an affective quality, but are not affects as such. Accordingly, such words cannot be found in Bradley and Lang’s (1999) Affective Norms for English Words (ANEW). Moreover, taking a closer look at German dictionaries for emotion or sentiment analysis such as the Berlin Affective Word List Reloaded (BAWL-R) (Võ et al., 2009) or Affective Norms for German Sentiment Terms (ANGST) (Schmidtke et al., 2014), they contain only a few words suited to describing the affective qualities of atmospheres. For instance, words such as sad, anger, aggression, and anxiety describe emotions, but not atmospheres. In addition, many words included in BAWL-R and ANGST, like candidacy, flamingo, or horoscope, are not adequate to express affective atmospheres that are prevalent in organizations. Consequently, and since behavior and coexistence in organizations are driven by shared rather than private emotions (Julmi, 2017b), dictionaries for the analysis of emotions and sentiments cannot be transferred one-to-one to the analysis of atmospheres in organizations. It is therefore not surprising that a key focus of research on atmospheres is to treat them as distinct objects of knowledge, related to but not equated with affect or emotions (Anderson, 2009; Schmitz et al., 2011; Griffero, 2017; Brown et al., 2019; Trigg, 2020).

Against this background, the aim of our article is to develop a dictionary for German affective norms for atmospheres in organizations (GANAiO). The development of GANAiO fills an important gap in organizational research, offering researchers a novel tool to explore how atmospheres are experienced and managed within organizations. By advancing the study of atmospheres, GANAiO provides a new avenue for organizational research, potentially leading to practical strategies for fostering better workplace environments.

We structure our article as follows: First, we provide a brief overview on related work on CATA, and particularly the dictionary approach. Second, we introduce the circumplex model of affective atmospheres, which serves as our theoretical foundation. Third, we explain the method used for developing GANAiO. Fourth, we present and discuss the results and limitations of our dictionary before concluding the article.

## Related work

Our research to develop GANAiO is closely linked to the analysis of sentiment and emotion. While sentiment analysis, in short, aims to determine the writer’s attitude towards a particular subject of interest which can be positive, negative, or neutral, emotion analysis aims to capture emotion in text such as anger, fear, surprise, or happiness (Lehmann et al., 2017). To extract people’s attitude or emotions from big data such as customer reviews or entries in social media, two main approaches have been established in research: the machine learning approach and the dictionary approach. On the one hand, originating from computer science, the machine learning approach requires training data to categorize text. On the other hand, the more common approach in the social sciences is dictionaries, which are a stock of terms that label words (Bonta et al., 2019), following either a categorical or dimensional model. The categorical model groups words with similar connotations into categories. This enables one to identify relevant text passages in large and unstructured datasets as well as to capture the tone of the text (Hossfeld & Wolfslast, 2022). The dimensional model labels words along different dimensions. The most common ones are the pleasure-displeasure dimension (valence), in which words are labeled as positive, neutral or negative, and the arousal–sleepiness dimension (arousal), which measures the intensity of emotion provoked by a stimulus from excited to calm (e.g., Bradley & Lang, 1994). The categorical and dimensional models can complement each other (Stevenson et al., 2007; e.g., Calvo & Mac Kim, 2013).

So far, scholars have developed several dictionaries for emotion and sentiment analysis. For example, Stevenson et al. (2007) and Briesemeister et al. (2011) attribute words to the emotion categories happiness, anger, fear, disgust, and sadness. Bradley and Lang (1999) developed Affective Norms for English Words (ANEW) to provide standardized materials for the study of emotions. In addition to valence and arousal, they rate many words along the dimension dominance, which assesses the degree to which someone feels in control (high dominance) or controlled (low dominance). The ANEW has been adapted to many different languages (Redondo et al., 2007; Soares et al., 2012; Moors et al., 2013; Warriner et al., 2013; Monnier & Syssau, 2014; Montefinese et al., 2014; Schmidtke et al., 2014; Imbir, 2015; Stadthagen-Gonzalez et al., 2017). Several of these adaptions additionally integrate the dimension of imageability (e.g., Schmidtke et al., 2014). This property is used to understand the relations between language and emotion and clarifies the degree of a word’s concreteness or abstractness in terms of emotion. The BAWL-R (Võ et al., 2009) is well established in German-speaking countries and rates over 2900 words along the dimensions valence, arousal, and imageability. It moreover serves as a reference model for further dictionaries such as ANGST (Schmidtke et al. 2014).

Although different dictionaries use different dimensions, they all consider the dimensions of pleasure–displeasure and arousal–sleepiness. These dimensions are considered as the two core dimensions of affective experiences (Russell, 2003) in the literature. The extent to which these dimensions are relevant for the study of atmospheres is the subject of the following section.

## Theoretical foundation

Like emotions, atmospheres are essentially affective phenomena (Schmitz et al., 2011). Russell and Pratt (1980, p. 311) try to provide a general description of affect and define it “as emotion expressed in language.” They further describe the affective quality of the environment as “the emotion-inducing quality that persons verbally attribute to that place” (Russell & Pratt, 1980, pp. 311–312). In their empirical model, they conceptualize the affective meaning of words that describe the quality of the environment as a two-dimensional bipolar space. This space is defined by the two orthogonal bipolar dimensions of pleasure–displeasure and arousal–sleepiness. Within this space, they defined eight variables arranged in a circular order. These variables, set at specific angles, are pleasant (0°), exciting (45°), arousing (90°), distressing (135°), unpleasant (180°), gloomy (225°), sleepy (270°), and relaxing (315°, positioned 45° from pleasant). This means, for example, that exciting is a combination of pleasant and arousing, while gloomy is classified as a combination of unpleasant and sleepy.

**Fig. 1:**
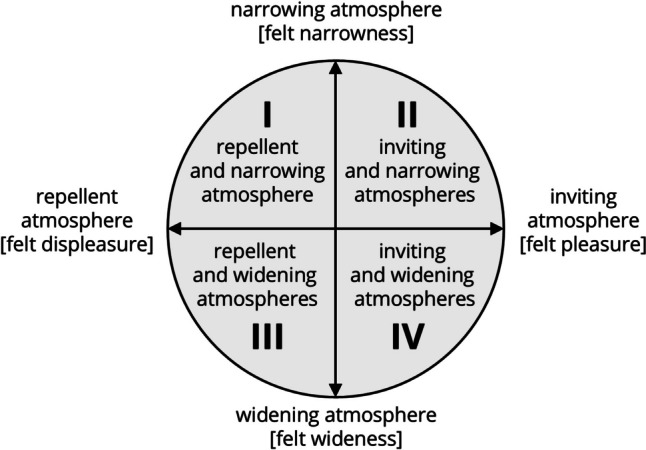
A circumplex model of affective atmospheres (Julmi, 2022)

Julmi (2015, 2017a, 2022, 2024) follows these arguments and understands the affective meaning of the environment as the affective atmosphere of this environment. He describes the affective quality of atmospheres in a circumplex model along the dimensions of pleasure/displeasure and narrowness/wideness (see Fig. 1). In particular, he distinguishes atmospheres based on the two dimensions of (1) inviting/repellent atmospheres and (2) narrowing/widening atmospheres. Inviting atmospheres exert an affective pull, encouraging individuals to linger or enter, while repellent atmospheres create a sense of urgency to leave or avoid a particular environment. Narrowing atmospheres have a concentric tendency from the spatial environment to one’s own felt presence, distinctly defining the spatially felt “here.” In contrast, widening atmospheres have an eccentric tendency, directed from one’s own felt presence into the spatial environment, so that one feels detached in the wideness from the spatially felt “here.” These dimensions give rise to four distinctive ideal atmosphere types: repellent and narrowing atmospheres, inviting and narrowing atmospheres, repellent and widening atmospheres, and inviting and widening atmospheres.

Julmi’s circumplex model of affective atmospheres correlates to the model from Russell and Pratt (1980) insofar as the inviting/repellent dimension corresponds to the pleasant/unpleasant dimension, and the narrowing/widening dimension corresponds to the arousing/sleepy dimension (for a discussion of the relationship between the two approaches see Julmi, 2022, pp. 16–18).

In conclusion, both approaches indicate that the dimensions of pleasure–displeasure and arousal–sleepiness are significant for describing the quality of the environment and hence for describing atmospheres. While valence and arousal are primary dimensions underlying the perception and experience of emotion, our study specifically targets the concept of atmospheres within organizational contexts. Atmospheres are not merely a reflection of affect but encompass the broader environmental qualities that characterize an organizational setting. By gathering data on norms related to organizational atmospheres, we aim to capture these nuanced environmental qualities that are pivotal in shaping organizational behavior and employee experiences. In this context, we aim to conceptualize GANAiO as a tool for dictionary-based content analysis along these dimensions, with the goal of enhancing research on organizational atmospheres and contributing to theory building.

## Method

### Corpus creation

Deng et al. (2019) and Schwartz and Ungar (2015) recommend building a dictionary on data that represent the subject of interest. Following this line of argument, we base our research on *kununu* data. Employer reviews on *kununu* provide rich verbal descriptions of work atmospheres (kununu, 2023).

*kununu* systematically administers surveys to employees across diverse categories, encompassing corporate culture, diversity, working environment, and career & salary. In addition to responding to structured questions, employees can articulate their perspectives on positive and negative aspects of their employer, offering valuable insights for potential improvements. Our comprehensive analysis meticulously considers feedback pertaining to corporate culture, specifically focusing on aspects such as the work atmosphere, communication, team spirit, leadership behavior, work–life balance, and interesting tasks. Furthermore, we pay special attention to comments regarding working conditions. We regard these comments as integral to our research, acknowledging their pivotal role in shaping the overall work atmosphere (Radermacher & Herdejürgen, 2022). Our data included a total of 756,065 comments from 2010 to 2019.

For data processing, we analyzed the respective comments in MAXQDA and determined word frequencies. Stop lists were applied and the words were lemmatized to avoid word duplication and words without substance (MAXQDA, 2023b). For our initial corpus, we considered words that were mentioned at least 10 times. In accordance with Deng et al.'s (2019) recommendation, validation of the dictionary entries was conducted through the expertise of subject matter specialists. In our capacity as experts in the field, we thoroughly revised the corpus, omitting words deemed inappropriate for capturing affective atmospheres. The resulting finalized corpus comprises a total of 5956 words.

### The categorical approach

#### Materials

According to Deng et al. (2019), one can derive categories from theory, data, or both. Given the current scarcity of research on atmospheres in organizations, with a notable absence of explicit differentiation in terms of, for instance, types or categories, atmospheric categories cannot be grounded solely in theory. Hence, we followed a data-driven, inductive approach to build our categories, which makes them more exploratory in nature (Deng et al., 2019). Given that people often speak about atmospheres as naturally as they do about the weather and intuitively name different types of atmospheres (Julmi, 2015; Gugutzer, 2020), we leveraged our dataset to gain preliminary insights into how employees describe various organizational atmospheres. We systematically analyzed the dataset, focusing on comments containing the term “atmosphere.” This initial exploration revealed several atmospheres, such as anxiety [Angst-], train station [Bahnhofs-], office [Büro-], call center [Callcenter-], pressure [Druck-], elbow [Ellbogen-], family [Familien-], collective [Gemeinschafts-], conversation [Gesprächs-], open-plan office [Großraumbüro-], competitive [Konkurrenz-], surveillance [Überwachung-], learning [Lern-], mistrust [Misstrauens-], start-up [Start-up-], team [Team-], trust [Vertrauens-], and feel-good atmosphere [Wohlfühlatmosphäre]. To further refine these categories, we conducted a keyword-in-context analysis using MAXQDA software (MAXQDA 2023a). Our analysis revealed a spectrum of descriptors used by employees to characterize atmospheres in organizations. These descriptors included terms such as *pleasant* [angenehm], *tense* [angespannt], *anxious* [bedränglich], *cool* [cool], *relaxed* [entspannt], *familiar* [familiar], *friendly* [freundlich], *collegial* [kollegial], *concentrated* [konzentriert], *laid-back* [locker], *professional* [professionell], *awesome* [super], *great* [toll], *toxic* [toxisch/vergiftet], and *trusting* [vertrauensvoll], or such as *good* [gut] and *bad* [schlecht] or *positive* [positive] and *negative* [negative].

We argue that terms such as “good” and “bad” do not provide meaningful differentiation for this study, as they can be easily categorized along the dimensions of valence and arousal. Similarly, many of the adjectives, such as tense, anxious, cool, relaxed, concentrated, laid-back, professional, and awesome, predominantly describe emotional states or characteristics that can manifest across various organizational contexts, rather than representing specific organizational atmospheres. Furthermore, these descriptors lack a clear frame of reference in terms of content, making it challenging to assign them to a particular type of atmosphere.

We also excluded categories like “train station” and “office” atmospheres, as they describe physical or environmental contexts rather than affective atmospheres that shape organizational interactions. These terms are more indicative of spatial settings rather than distinct atmospheres within organizations. Similarly, categories like “conversation” and “learning” atmospheres were not considered further, as they lack a specific and representative meaning within an organizational context. As “train station” and “call center” atmospheres were often mentioned alongside the “open-plan office,” we combined these terms into the broader category of “open-plan office atmosphere.” The distinction between “elbow” and “competitive” atmospheres also proved difficult to maintain, leading to their unification under the “competitive atmosphere” category. Similarly, categories like “control,” “mistrust,” and “surveillance” atmospheres were combined into a single “surveillance atmosphere,” as were “collegial” and “team” atmospheres, and “toxic” and “poisonous” atmospheres.

By refining these categories and merging overlapping ones, we identified 11 distinct categories of atmospheres: toxic, anxiety, family, competitive, open-plan, start-up, team, surveillance, trust, feel-good, and pressure atmospheres.

#### Participants and procedure

In the subsequent phase, the terms within the corpus were categorized through a web survey conducted via Unipark. This process was an integral component of the “Atmospheres in Organizations” course at the Faculty of Business Administration and Economics held in winter semester 2023/2024. A total of 32 students, consisting of 17 female and 15 male participants, actively took part in the web survey. Each participant was asked to determine whether each word should be assigned to none, one, or multiple atmosphere categories. In recognition of their contributions, students received 10 ECTS for the entire course, including the survey, the writing of a seminar paper, and giving oral presentations. A word was assigned to a category if it received assignments from at least 50% of the participants.

### The dimensional approach

#### Participants

A total of 1303 people participated in our survey on the dimensional approach, each of whom was asked to evaluate 500 words in terms of either valence or arousal. However, only 850 participants completed the survey, of whom 322 were male, 515 were female, and three were diverse. Ten participants did not disclose their gender. The loss of 453 participants is attributed to two main factors: the length of the survey, which averaged 43 minutes and 28 seconds, with a median of 37 minutes and 53 seconds, and the perceived monotony associated with evaluating 500 words using the Self-Assessment Manikin (SAM). The study, not restricted to a specific target demographic except for fluency in German, attracted a heterogeneous participant pool. In terms of age distribution, 32 participants were younger than 20, 353 fell within the 21–30 age range, 224 were between 31 and 40, 125 between 41‍ and 50, 88 between 51 and 60, and 28 older than 60. Furthermore, 294 of the participants were enrolled full-time and 334 part-time at a university, spanning multiple disciplines. In appreciation of their contribution, each participant had the opportunity to receive a gift voucher worth €15.

#### Procedure

**Fig. 2:**
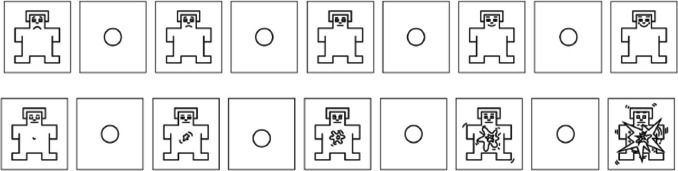
The Self-Assessment Manikin (SAM) (Betella & Verschure, 2016), top row: valence, lower row: arousal

We closely followed the methodology for the dimensional approach of dictionaries such as the ANEW, the BAWL-R (Võ et al., 2009), and ANGST (Schmidtke et al., 2014). We rated the dimensions of valance and arousal along a nine-point scale to measure both valence (−4 for very negative, 0 for neutral, and +4 for very positive words) and arousal (1 for low arousal and 9 for high arousal). The assessment of the two dimensions was carried out by means of the SAM, a nonverbal pictographic assessment technique developed by Bradley and Lang (1994) to measure pleasure, arousal, and dominance (see Fig. 2), and by means of a web survey. To avoid transfer effects, the dimensions were surveyed separately (Schmidtke et al., 2014). Following instructions closely aligned with Bradley and Lang (1999) and Moors et al. (2013), and after three practice trials, each participant rated 500 randomly assigned words in the context of work atmospheres (Soares et al., 2012; Schmidtke et al., 2014). For the processing and analysis of data, we used Python version 3.11.5 and the matplotlib library to generate scatter plots.

The cleaning of the data was divided into two main steps. At the participant level, we excluded evaluations with low variability from the analysis. Specifically, we identified and removed ratings with a standard deviation less than 1, which occurred when participants provided the same rating for all words. As a result, we removed 32 participants (20 male, 10 female, two not specifying their gender), leaving us with 818 valid responses: 302 male, 505 female, three diverse, and eight not specifying their gender. At the word level, statistical outliers were then removed using the interquartile range (IQR), which represents the difference between the first quartile (Q1) and the third quartile (Q3). The bounds for outliers are defined by setting the multiplication factor to zero, resulting in bounds of [Q1, Q3]. As a result, all values outside this range were eliminated from the dataset. In a final step, we calculated the mean and the standard deviation for each word and for both dimensions.

## Results and discussion

### Ratings in the valence-arousal space

**Fig. 3:**
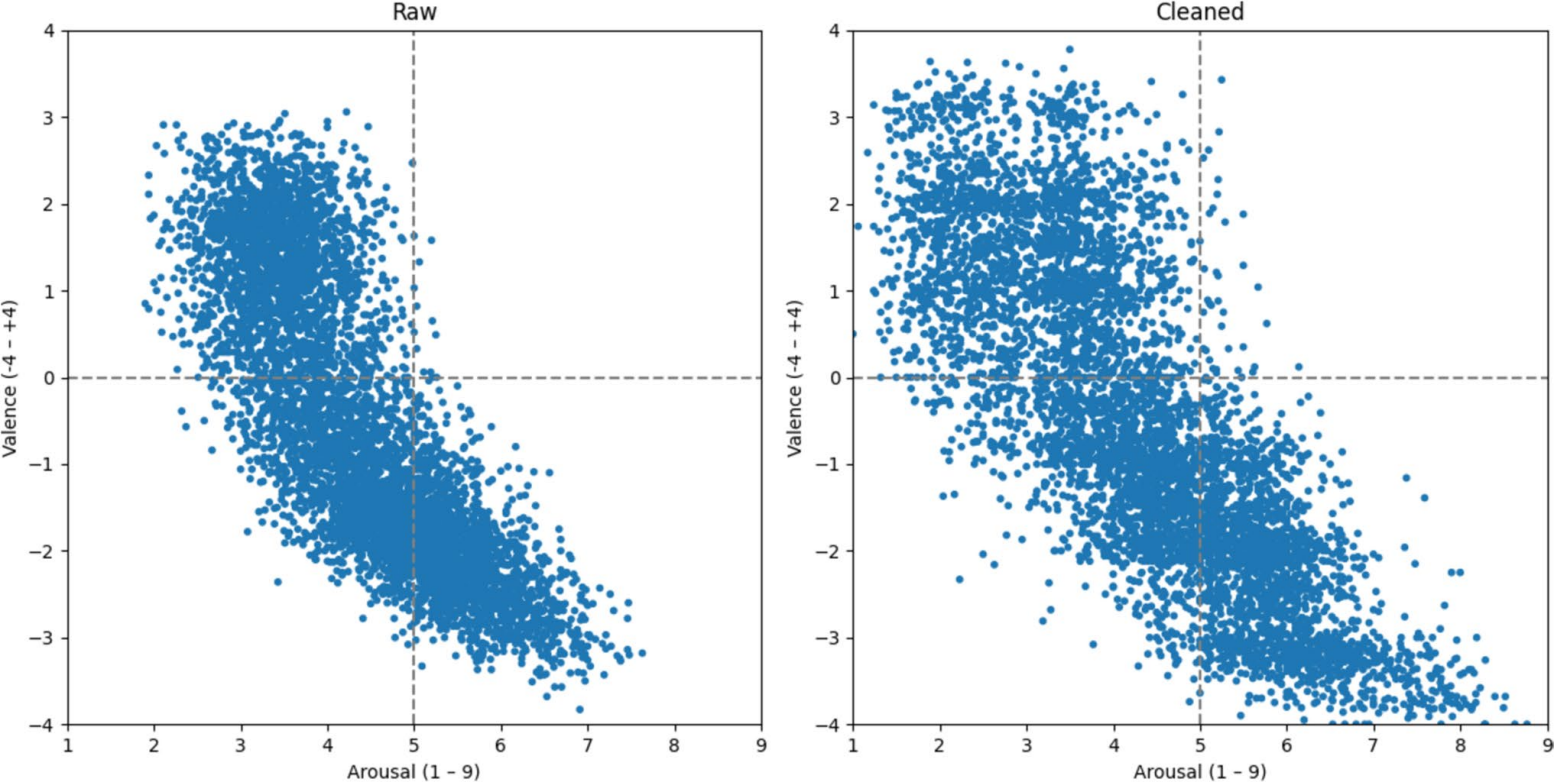
Average ratings per word in the valence-arousal space

Our results of the dimensional study are partly consistent with comparable studies and partly somewhat unexpected. Figure 3 shows the ratings of the 5956 words in the valence–arousal space as scatter plots, with the raw data on the left and the trimmed data on the right. In the raw data, participants with low variance have already been excluded, but statistical outliers at the word level have not yet been removed.

After data cleaning, we observed a significant increase in data dispersion compared to the raw dataset. The removal of statistical outliers reduced the concentration of data points, resulting in a more heterogeneous distribution. Without these extreme values, the cleaned dataset showed a broader spread in the valence–arousal space, making the increased scatter more visually apparent.

Interestingly, the distribution shown in Fig. 3 apparently does not fit the typical boomerang shape found by Bradley and Lang (1999) and others. In line with other research (e.g., Redondo et al., 2007; Montefinese et al., 2014; Stadthagen-Gonzalez et al., 2017), unpleasant words are associated with heightened levels of arousal. For instance, the words *terror* [Terror], *war* [Krieg], *tyrant* [Tyrann], and *inhuman* [menschenverachtend] were each rated with a valence of −4 with corresponding arousal levels of 7.78, 8.77, 8.29, and 8.28. Additionally, many words with an intermediate valence were also rated with an intermediate level of arousal (e.g., *everyday life* [Alltag], valence (v): 0; arousal (a): 3.38; *dress code* [v: −0.5; a: 4.45; Dresscode]). However, we find that pleasant words do not have high levels of arousal (e.g., *peace* [v: 3.36; a: 1.74; Frieden], *harmony* [v: 3.28; a: 2.19; Harmonie]). Although it is common for positive words to evoke lower levels of arousal than negative words (Võ et al., 2009; Kuppens et al., 2013; Moors et al., 2013; Warriner et al., 2013; Schmidtke et al., 2014; Stadthagen-Gonzalez et al., 2017), the positive words in this study appear to trigger even lower arousal levels than those reported in previous research.

To confirm our first impressions of the scatter plot, we calculated the mean arousal and valence ratings (and standard deviations) for negative (v: ≥ −4 to ≤ −1), neutral (v: > −1 to ≤ +1), and positive (v: > +1 to ≤ +4) words and looked for differences regarding gender. Table 1 shows the results. These confirm that positive words, in our dataset, as opposed to related research, have a lower arousal than neutral and negative words. We consider three possible explanations.

**Table 1:** Mean arousal and valence ratings (± *SD*) for the total sample, males, and females

	Arousal ratings		Valence ratings	
	Mean	*SD*	Mean	*SD*
Total sample	4.35	1.12	−0.57	0.76
Males	4.36	0.97	−0.45	0.65
Females	4.31	1.10	−0.64	0.72
All subjects				
Negative	5.44	1.10	−2.22	0.78
Neutral	3.73	1.14	−0.07	0.73
Positive	2.99	1.15	1.95	0.77
Males				
Negative	5.19	0.96	−2.01	0.66
Neutral	3.92	0.96	0.02	0.63
Positive	3.36	0.98	1.92	0.67
Females				
Negative	5.50	1.08	−2.34	0.73
Neutral	3.61	1.11	−0.04	0.70
Positive	2.77	1.11	2.06	0.73

First, emotions and atmospheres are two distinctively different constructs. Thus, the valence–arousal ratings in emotion research may not be transferable one-to-one to atmospheres. Moreover, we explicitly instructed the participants to rate the words in the context of work atmospheres. According to the observation that organizational settings often emphasize stability and control, high positive arousal states might be suppressed (Barsade & Gibson, 2007). Hence, one could reasonably infer that elevated positive arousal in the work context seems to be less common compared to both leisure time and pleasant emotional situations.

Second, unlike previous studies (e.g., Bradley & Lang, 1999; Redondo et al., 2007; Schmidtke et al., 2014; Stadthagen-Gonzalez et al., 2017), our participants came from diverse backgrounds, not just psychology students familiar with the SAM and the valence–arousal space. However, despite a heterogeneous field of participants, Imbir (2015) and Warriner et al. (2013) could identify a U-shaped curve, suggesting that differences are not solely demographic. We closely aligned our methodology with related research, minimizing methodological discrepancies. Notably, our word corpus is derived from *kununu* data. *kununu* data may have unique features and nuances specific to workplace atmospheres that are not captured in general language sentiment lexicons like BAWL-R and ANEW. However, the domain specificity in GANAiO is important for accurately capturing the intricacies of organizational atmospheres. Thus, differences in data sources may contribute to variability in results.

Third, cultural and linguistic differences may influence arousal ratings, with studies noting greater variability in arousal than valence ratings across languages (Redondo et al., 2007; Warriner et al, 2013; Montefinese et al., 2014; Schmidtke et al., 2014). For instance, positive words in German elicit lower arousal compared to English ratings. Despite this, Võ et al. (2009) and Schmidtke et al. (2014) successfully replicated the boomerang shape for German words. We posit that, apart from atmospheres differing from emotions, the work context plays a pivotal role in the context of low arousal ratings for positive words. Future research could explore and replicate our study on atmospheres in different languages, considering both work and non-work contexts.

In order to assess the internal consistency of participants’ valence and arousal ratings, we applied the split-half method. For this purpose, we divided participants into two groups based on their order of participation. Mean ratings were computed for each unique word. Our analyses revealed a highly significant bivariate Pearson correlation between mean valence ratings (*r* = 0.923, *p* < 0.001) and moderate correlation for arousal (*r* = 0.683, *p* < 0.001). We adjusted the reliability of valence to 0.960 and arousal to 0.812 using the Spearman–Brown formula. These findings suggest strong internal consistency in raters’ evaluations of both valence and arousal dimensions, underscoring their reliability and stability in this context. The higher reliability observed for valence compared to arousal ratings aligns consistently with findings from prior studies in similar domains, emphasizing the robustness of our results (Ekkekakis, 2013; Warriner et al., 2013; Monnier & Syssau, 2014; Stadthagen-Gonzalez et al., 2017).

**Fig. 4:**
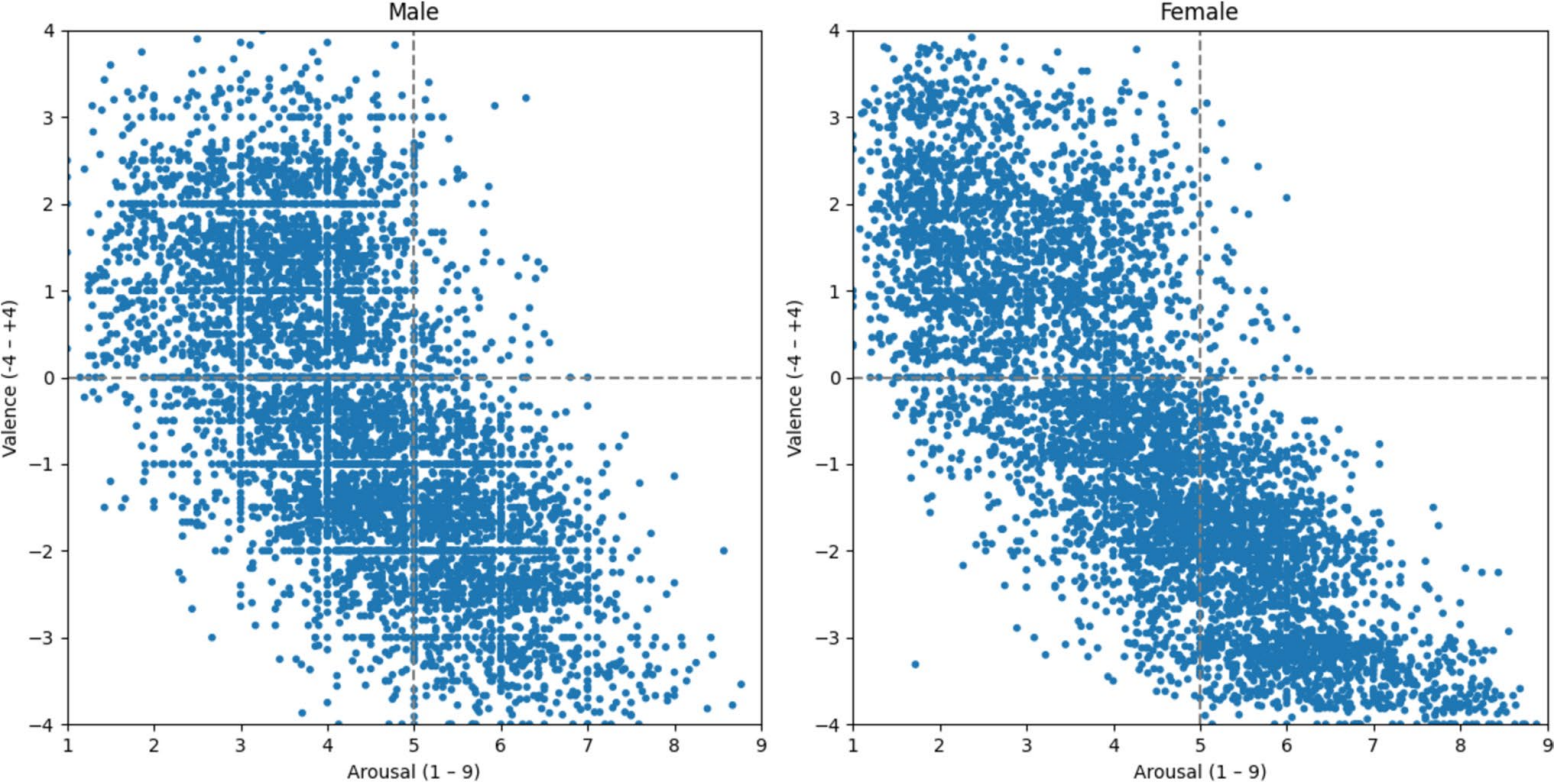
Average male and female ratings per word in the valence-arousal space

Taking a closer look at gender differences in our ratings (see Table 1 and Fig. 4), the data distribution of male ratings, as opposed to the female ratings, appears to display a grid-like pattern along the integers. We argue that this pattern is likely attributable to the lower number of male ratings (515 female compared to 322 male) and the removal of participants (10 female compared to 20 male). Firstly, the presence of fewer male ratings inherently results in a sparser distribution of data points along the rating scale. Consequently, this can lead to a more pronounced pattern in the data distribution, characterized by distinct clusters of ratings. Secondly, the removal of participants further exacerbates this effect. With participants eliminated, the variability of ratings per word in the male dimension is reduced. This increases the probability that the ratings for a particular word will remain constant across different evaluations and that the average value forms a whole number. This phenomenon contributes to the emergence of the observed grid-like pattern in the data distribution of male ratings.

Contrary to the stereotype that women express emotions with greater intensity than men (Montefinese et al., 2014), our data, in line with other research (Redondo et al., 2007; Monnier & Syssau, 2014), reflect a general consensus between gender in the ratings of both affective dimensions, as depicted in Fig. 4. Nonetheless, Monnier and Syssau (2014) and Soares et al. (2012) find that females rate pleasant words as more exciting than men. This contrasts with our results, where male participants rate positive words with an average arousal rate of 3.36, surpassing the female average arousal rate of 2.77 for pleasant words. Conversely, this pattern is reversed for negative words: women exhibit an average arousal rate of 5.50 for unpleasant words, compared to 5.19 for men. Regarding valence ratings, our data reveal that females rate pleasant words more positively and unpleasant words more negatively than men. These findings align with the literature (Monnier & Syssau, 2014), suggesting that females are more inclined to rate words at the extremes of affective scales. Our results imply that women may perceive negative stimuli more intensely than men, while men may perceive positive stimuli more intensely than women.

**Fig. 5:**
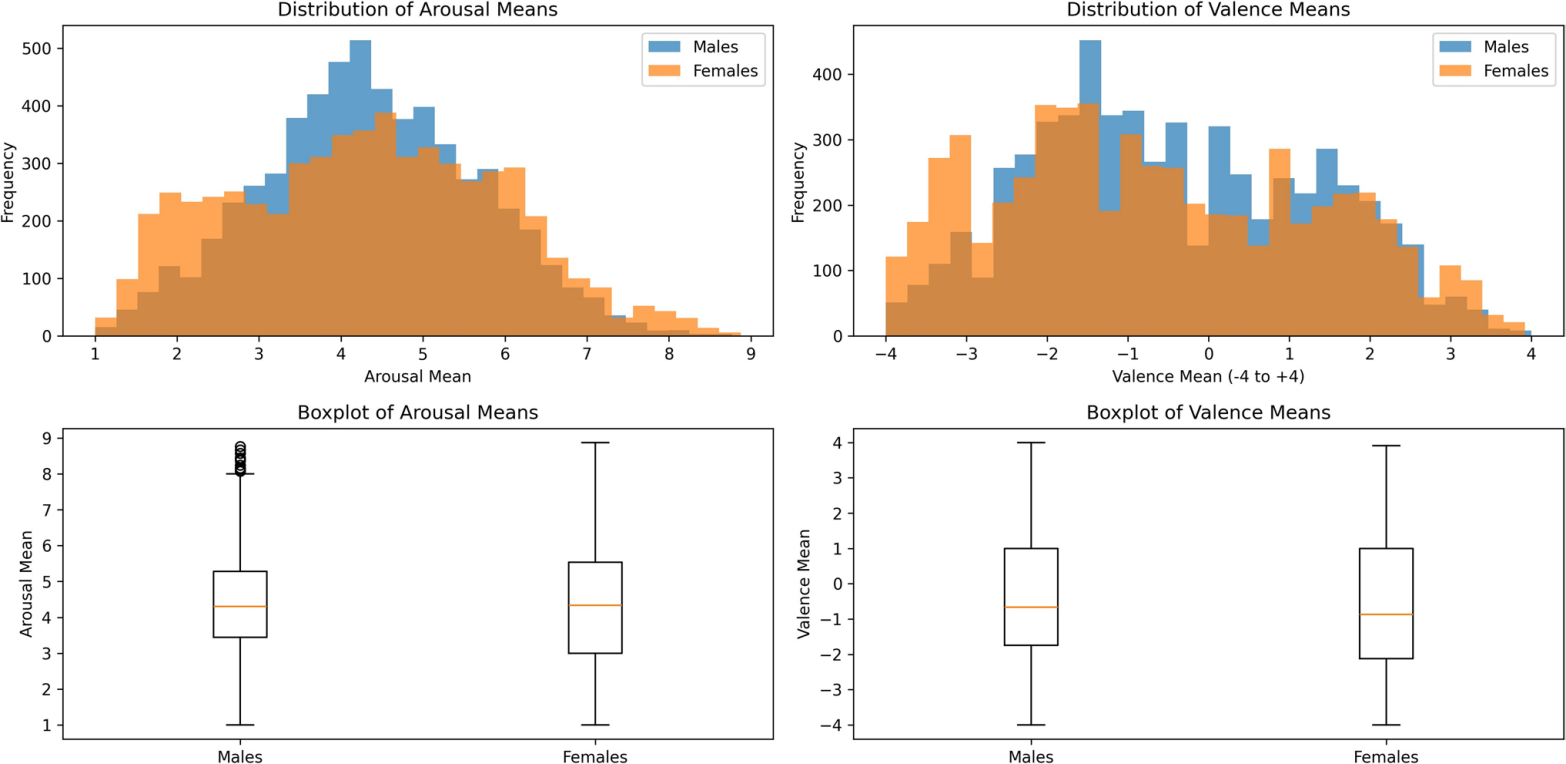
Distribution of arousal and valence mean values for males and females

To further examine gender differences, we conducted Welch’s *t*-tests on the mean values for both valence and arousal (see Fig. 5). To calculate the degrees of freedom (*df*), we used the Welch–Satterthwaite equation. The Welch’s *t*-test results for arousal indicate no statistically significant difference between genders regarding arousal (*t* = −1.72, *p* = 0.085, *df* = 11,460.39). In contrast, Welch’s *t*-test for valence reveals a highly significant difference (*t* = −5.65, *p* < 0.001, *df* = 11,736.07), indicating that males have a higher average valence than females. The distribution shown in Fig. 5 also indicates that females have a more diverse range of valence ratings, including both higher positive and more negative ratings than males. Overall, these results suggest that while there are no significant differences in arousal levels between genders, males and females exhibit significant differences in their average valence ratings.

We also used Levene’s test to compare the variances between male and female groups to determine whether there were significant differences in the variability of emotional responses. Levene’s test for arousal (Levene statistic = 22.58, *p* < 0.001) showed a significant difference in the variances between males and females. Specifically, we calculated the variability across different words for each group, which allowed us to determine the consistency of their emotional responses to different stimuli. The standard deviation of these word-specific arousal standard deviations was 0.36 for females and 0.39 for males, indicating slightly greater variability in the male group. Similarly, Levene’s test for valence (Levene statistic = 131.60, *p* < 0.001) also indicated a significant difference in variances, with males showing greater variability. The standard deviation of the word-specific valence standard deviations was 0.28 for females and 0.34 for males, suggesting that males exhibited more inconsistency in valence responses than females.

### Combining the categorical and dimensional approach

To scrutinize the distinct atmosphere types more comprehensively, we integrate the outcomes from both the dimensional and categorical approaches. This involves computing the average arousal and valence ratings for each type (see Table 2). Additionally, to enhance result visualization, we present scatter plots illustrating how words associated with each type are positioned in the valence–arousal space.

**Table 2:** Number of words, mean arousal, and valence ratings (± *SD*) for each type

		Arousal rating		Valence rating	
Type	Number of words	Mean	*SD*	Mean	*SD*
Feel-good	550	2.63	1.15	2.05	0.79
Family	113	2.71	1.24	1.89	0.83
Trust	234	2.99	1.18	2.21	0.74
Team	339	3.12	1.17	1.70	0.76
Start-up	143	3.21	1.18	1.45	0.75
Open-plan office	131	4.15	1.17	−0.94	0.74
Competitive	323	5.85	1.06	−2.27	0.79
Pressure-laden	387	5.40	1.09	−1.76	0.79
Surveillance	114	5.46	1.09	−2.11	0.82
Anxiety	289	5.88	1.15	−3.56	0.73
Toxic	784	6.17	1.11	−2.82	0.76

Of the 11 identified (non-exhaustive) types of atmospheres, most can be positioned in either the upper left or lower right quadrant, according to the scatter plots. The results in Table 2 confirm that there are similarities between the more pleasant and relaxed types of atmospheres and the ones which are more unpleasant and tense. Only the open-plan office atmosphere shows somewhat mixed results.

In the synopsis of the scatter plots, some of the types appear to give a rather similar picture. However, an analysis of the intersections between the word lists of the types shows that the types differ greatly in terms of quality. Table 3 shows the results for a disjoint set analysis for the toxic atmosphere, the anxiety atmosphere, the surveillance atmosphere, and the pressure-laden atmosphere. The majority of words are assigned to one atmosphere type only, with the anxiety atmosphere being a notable exception. The overlap between the toxic and the anxiety atmosphere seems to be the largest, with 100 joint words. These include, for example, *violence* [v: −3.80; a: 8.12; Gewalt], *shouting* [v: −3.59; a: 6.67; Gebrüll], *vicious cycle* [v: −3.23; a: 5.95; Teufelskreis], *to threaten* [v: −3.8; a: 7.42; bedrohen], or *dreadful* [v: −2.48; a: 6.64; schrecklich]. One possible reason for the high overlaps between the anxiety atmosphere and the other types is that anxiety often results from or accompanies a toxic or pressure-laden atmosphere. In such environments, as research shows, anxiety can be a consequence of direct threats, violence, and ongoing pressure and stress (Ganster & Rosen, 2013; Yip et al., 2020). This might explain why words describing anxiety frequently appear in other negative atmospheres. Furthermore, specific negative emotions and states experienced in these atmospheres can trigger similar linguistic expressions, leading to an overlap in the word lists. Despite the overlaps, only three words appear on all four lists: *bad* [v: −3.31; a: 4.72; schlecht], *demotivation* [v: −3.16; a: 5.37; Demotivation], and *to sanction* [v: −2.47; a: 6.52; sanktionieren]. On this basis, we argue that each negative type, despite their similarities, has its distinct qualities. Similar results are obtained when comparing the family atmosphere, the team atmosphere, the trust atmosphere, and the feel-good atmosphere. Here, the four types share a total of 20 words, including *togetherness* [v: 2.95; a: 2.04; Zusammengehörigkeitsgefühl], *humanity* [v: 3.19; a: 3.25; Menschlichkeit], *friendly* [v: 3.00; a: 1.81; freundlich], and *considerate* [v: 3.13; a: 1.85; rücksichtsvoll].

**Table 3:**
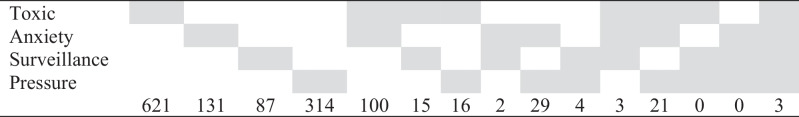
Disjoint set analysis for the toxic, anxiety, surveillance, and pressure-laden atmospheres

Due to their similar valence/arousal profiles, we consider it plausible that different types of atmospheres may exist simultaneously in an organization. For example, a family atmosphere may also be an atmosphere of trust and have a high level of team spirit. However, due to the qualitative differences, we only see this as a possibility, not a correlate. Moreover, the disjoint set analysis reveals notable differences that distinguish an anxiety from a surveillance atmosphere or a feel-good from a trust atmosphere. Relying solely on scatter plots does not yield clear conclusions about the prevalent atmosphere type in an organization. Nevertheless, leveraging our dictionary enables such distinctions and serves as a foundational step for more in-depth qualitative analyses. To explore these differences further, qualitative analysis is essential. The following section offers a brief overview of the identified types of atmospheres.

#### Feel-good atmosphere

**Fig. 6:**
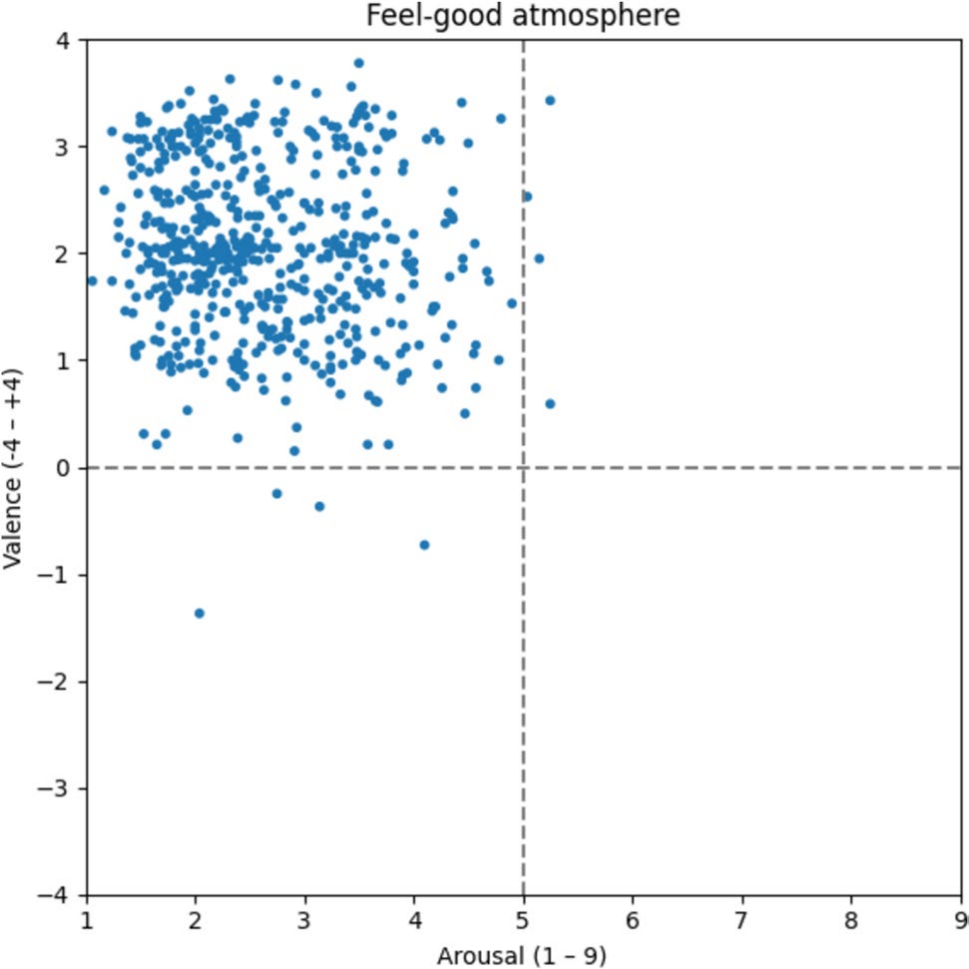
Feel-good atmosphere

In terms of the feel-good atmosphere (Fig. 6), we find that the majority of the 550 words are situated in the upper left quadrant, indicating a highly relaxed and pleasant ambiance. The average valence ratings (v: 2.05) reveal that the feel-good atmosphere is the second most positively rated and also characterized as the calmest (a: 2.63) type. At least 29 out of 32 participants associated words such as *contentment* [v: 2.43; a: 1.32; Zufriedenheit], *relaxation* [v: 2.79; a: 1.67; Entspannung], *joy* [v: 3.36; a: 3.52; Freude], and *regeneration* [v: 3.27; a: 2.48; Erholung] with a feel-good atmosphere.

#### Family atmosphere

**Fig. 7:**
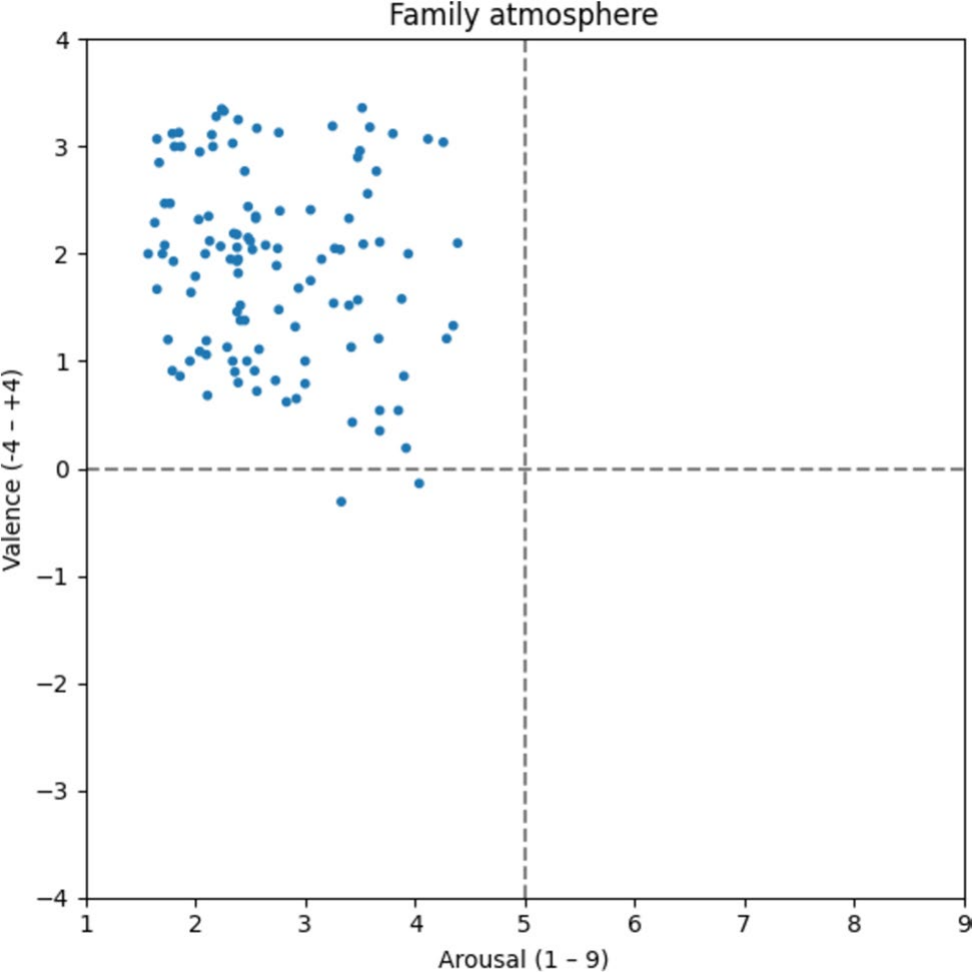
Family atmosphere

The family atmosphere (Fig. 7) appears to be assessed similarly to the feel-good atmosphere. However, with only 113 attributed words such as *familial* [v: 1.67; a: 1.65; familiär], *caring* [v: 2.35; a: 2.12; fürsorglich], *harmonize* [v: 2.47; a: 1.77; harmonieren], *heartiness* [v: 3.13; a: 2.76; Herzlichkeit], and *connectivity* [v: 2.40; a: 2.77; Verbundenheit], there seem to be fewer distinctive characteristics associated with the family atmosphere as opposed to the feel-good atmosphere.

#### Trust atmosphere

**Fig. 8:**
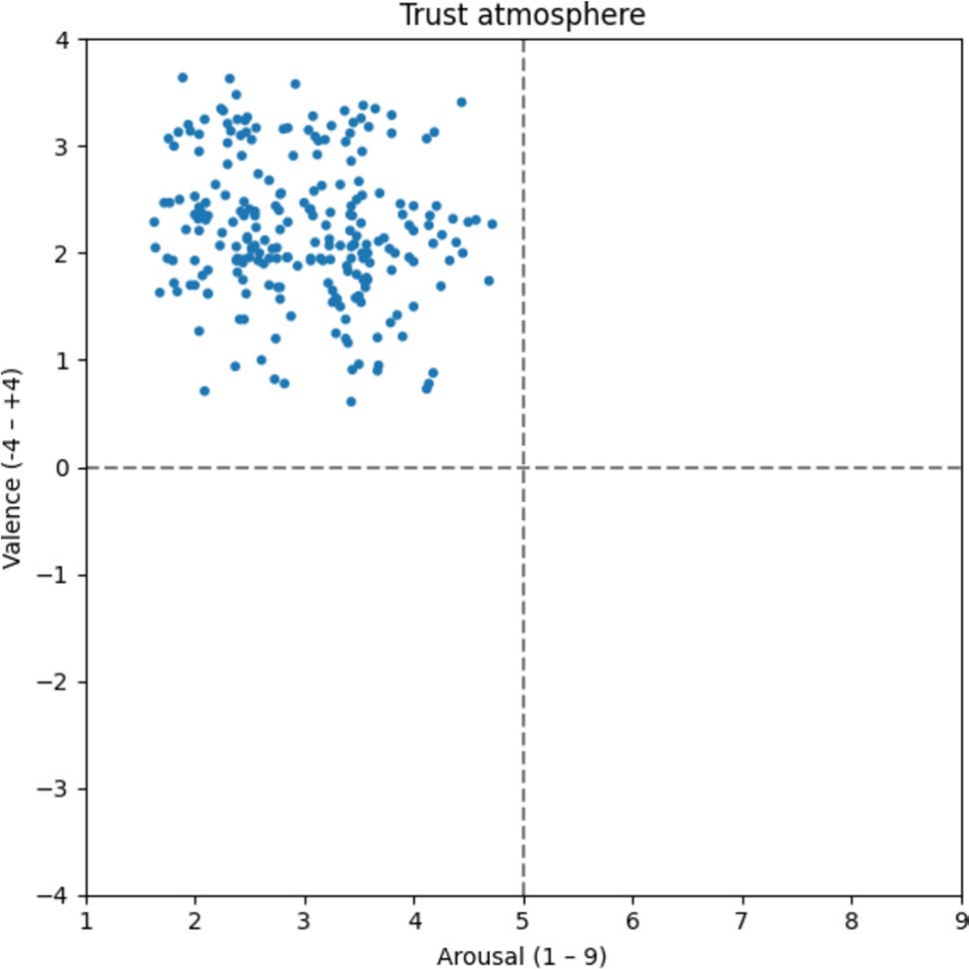
Trust atmosphere

The word list for the trust atmosphere paints a similar picture to the feel-good and family atmospheres (Fig. 8). *Sincerity* [v: 3.21; a: 2.30; Aufrichtigkeit] and *honesty* [v: 3.17; a: 2.56; Ehrlichkeit] as well as *sympathy* [v: 2.38; a: 2.06; Verständnis] and *respect* [v: 2.08; a: 3.45; respektieren] seem to be central for this typical atmosphere. Notably, the trust atmosphere boasts the highest average valence ratings (v: 2.21).

#### Team atmosphere

**Fig. 9:**
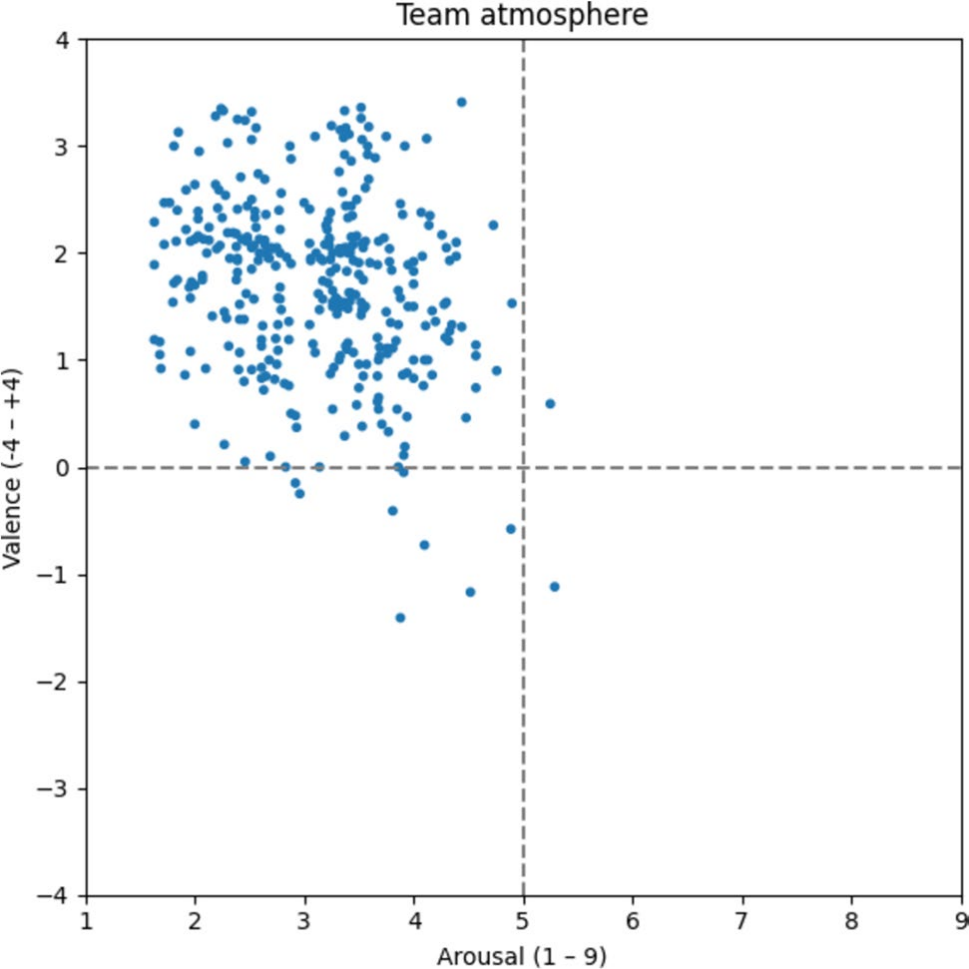
Team atmosphere

Most of the 339 words associated with the team atmosphere (Fig. 9) are in the upper left quadrant as well, which includes words such as *collaborative* [v: 2.11; a: 3.56; gemeinschaftlich], *collegiality* [v: 3.11; a: 3.41; Kollegialität], *team spirit* [v: 1.93; a: 3.45; Teamspirit], and *sense of belonging* [v: 2.44; a: 2.48; Zugehörigkeitsgefühl]. Based on the scatter plots and average ratings (v: 1.70; a: 3.12), we reason that a team atmosphere is also perceived as pleasant and calm.

#### Start-up atmosphere

**Fig. 10:**
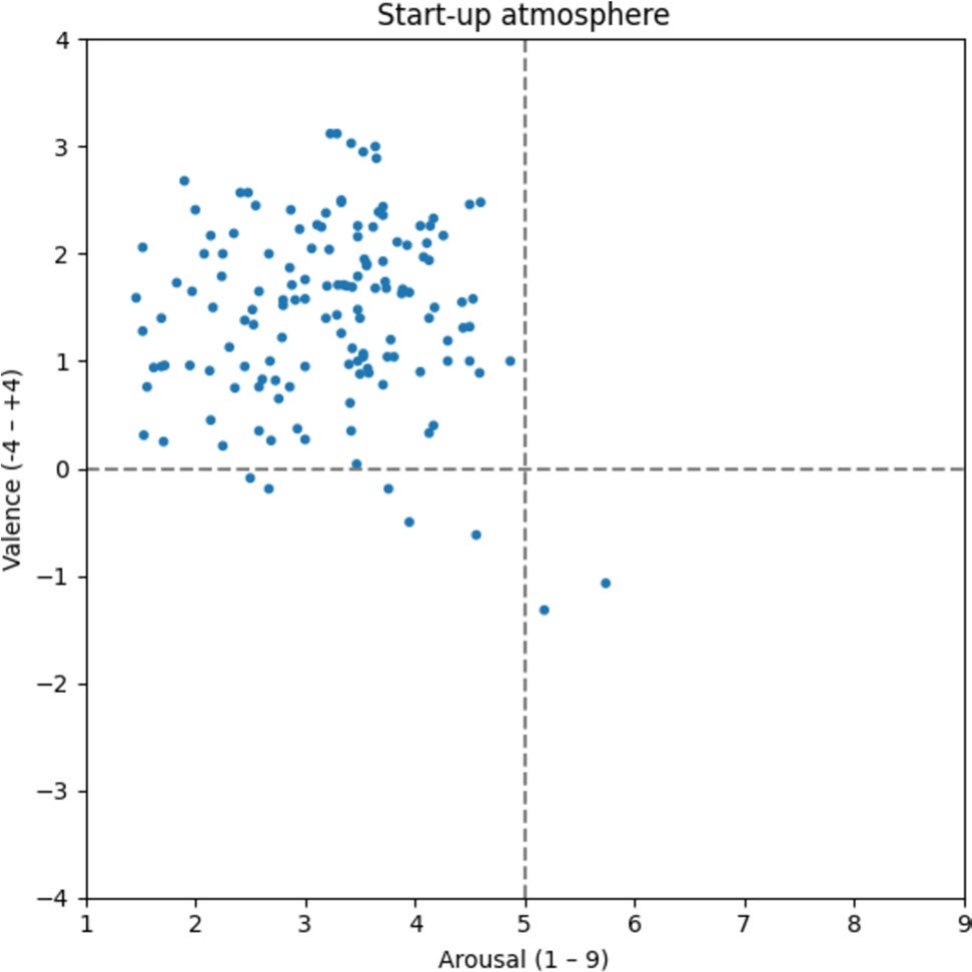
Start-up atmosphere

Compared to the aforementioned types of atmospheres, the start-up atmosphere (Fig. 10) is perceived as slightly less positive (v: 1.45) and somewhat more hectic (a: 3.21). However, most of the 143 words are also in the upper left quadrant. Aspects such as *agile* [v: 1.13; a: 2.31; agil], *innovative* [v: 1.7; a: 4.17; innovativ], *future-oriented* [v: 2.41; a: 2.87; zukunftsorientiert], *modern* [v: 1.4; a: 3.19; modern], and *visionary* [v: 6.58; a: 3.00; visionär], according to the ratings, seem to be important for a start-up atmosphere.

#### Open-plan office atmosphere

**Fig. 11:**
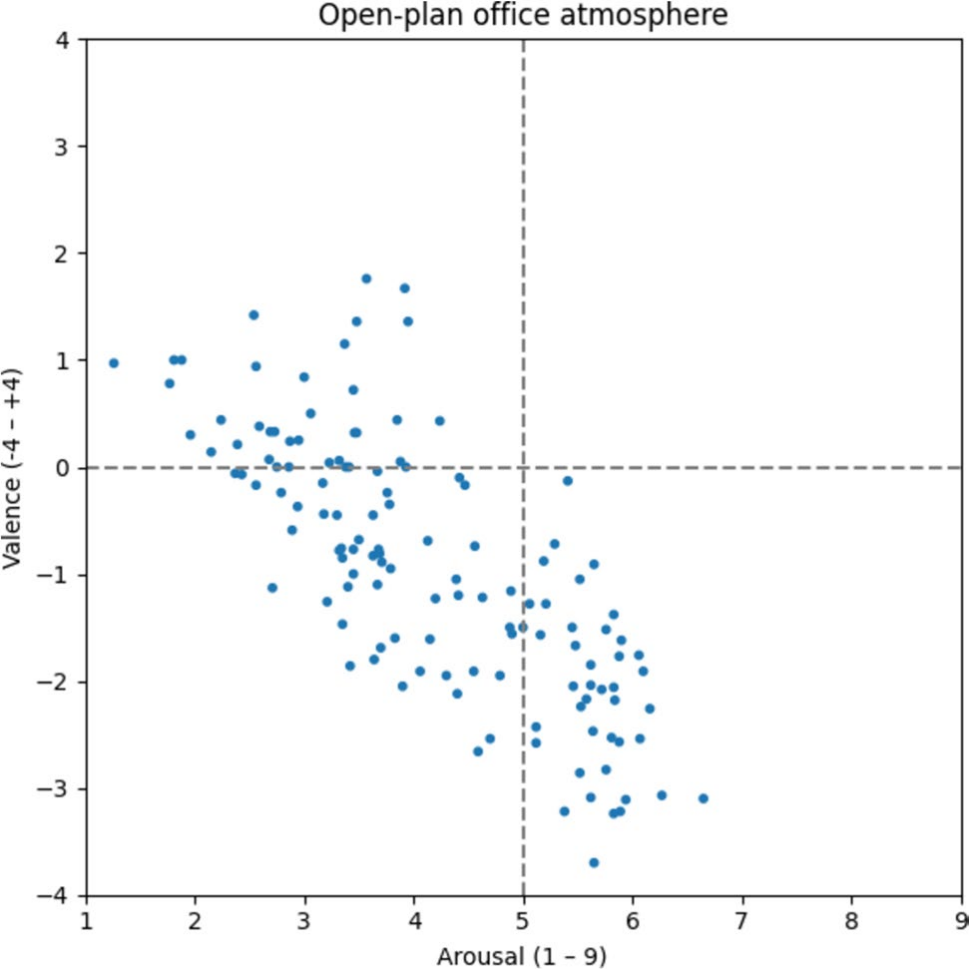
Open-plan office atmosphere

In contrast to the aforementioned predominantly positive atmospheres, a different picture emerges for the open-plan office atmosphere (Fig. 11). The 131 words assigned to this type are dispersed across all but the upper right quadrant. We therefore conclude that the open-plan office atmosphere itself is not inherently positive or negative, or calm or hectic, but encompasses mixed elements. This is also reflected in the average ratings (v: −0.94; a: 4.15), indicating that the open-plan office atmosphere serves as an interface between typical atmospheres that, on trend, can be categorized as either positive or negative. Words associated most with this type are *call center* [v: −1.38; a: 5.83; Callcenter], *noise level* [v: −1.52; a: 5.76; Lärmpegel], *shortage of space* [v: −1.62; a: 5.90; Platzmangel], and *dividing wall* [v: −0.78; a: 3.32; Trennwand].

#### Competitive atmosphere

**Fig. 12:**
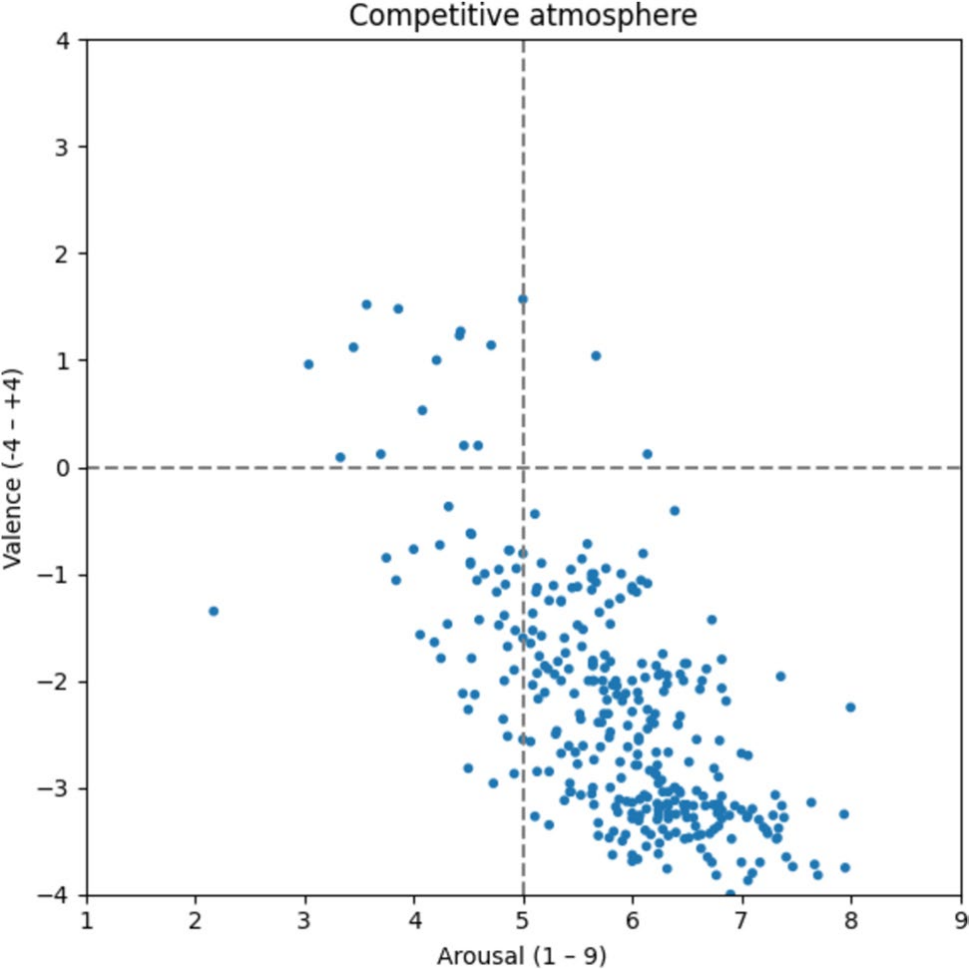
Competitive atmosphere

The majority of the 323 words belonging to the competitive atmosphere (Fig. 12) are in the lower right quadrant, indicating that this type is perceived as a tense, unpleasant atmosphere (v: −2.27; a: 5.85). Nevertheless, a few words such as *assertiveness* [v: 1.23; a: 4.42; Durchsetzungsfähigkeit], *ambitious* [v: 1.14; a: 4.71, ehrgeizig], and *competitive* [v: 1.12; a: 3.45, konkurrenzfähig] scatter in the neutral and positive zones. In that regard, *ego trip* [v: −3.36; a: 6.58; Egotrip], *competitor* [v: −0.81; a: 6.10; Konkurrent], *rivalry* [v: −1.81; a: 5.64; Rivalität], and *conflict* [v: −2.62; a: 5.96; Gegeneinander] seem to be major themes.

#### Pressure-laden atmosphere

**Fig. 13:**
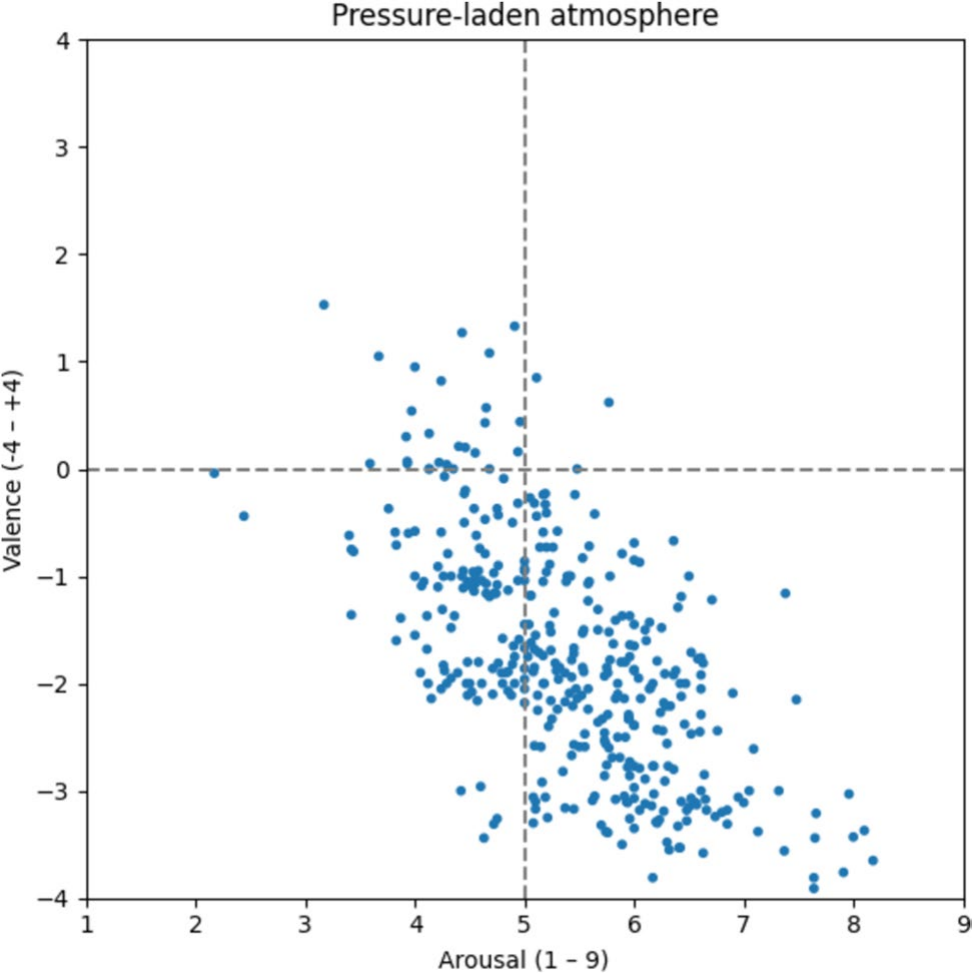
Pressure-laden atmosphere

By means of the scatter plot, the pressure-laden atmosphere (Fig. 13) can hardly be distinguished from the competitive atmosphere. However, based on the average ratings, it is perceived as slightly less hectic and negative (v: −1.76; a: 5.40). Nonetheless, due to the frequent categorization of 387 words such as *stress* [v: −2.00; a: 6.47; Stress], *overwork* [v: −3.56; a: 7.37; Arbeitsüberlastung], *urgency* [v: −0.09; a: 4.81; Dringlichkeit], *overload* [v: −2.89; a: 6.10; Überlastung], and *exhaustion* [v: −3.32; a: 5.70; Erschöpfung], we identify qualitative differences.

#### Surveillance atmosphere

**Fig. 14:**
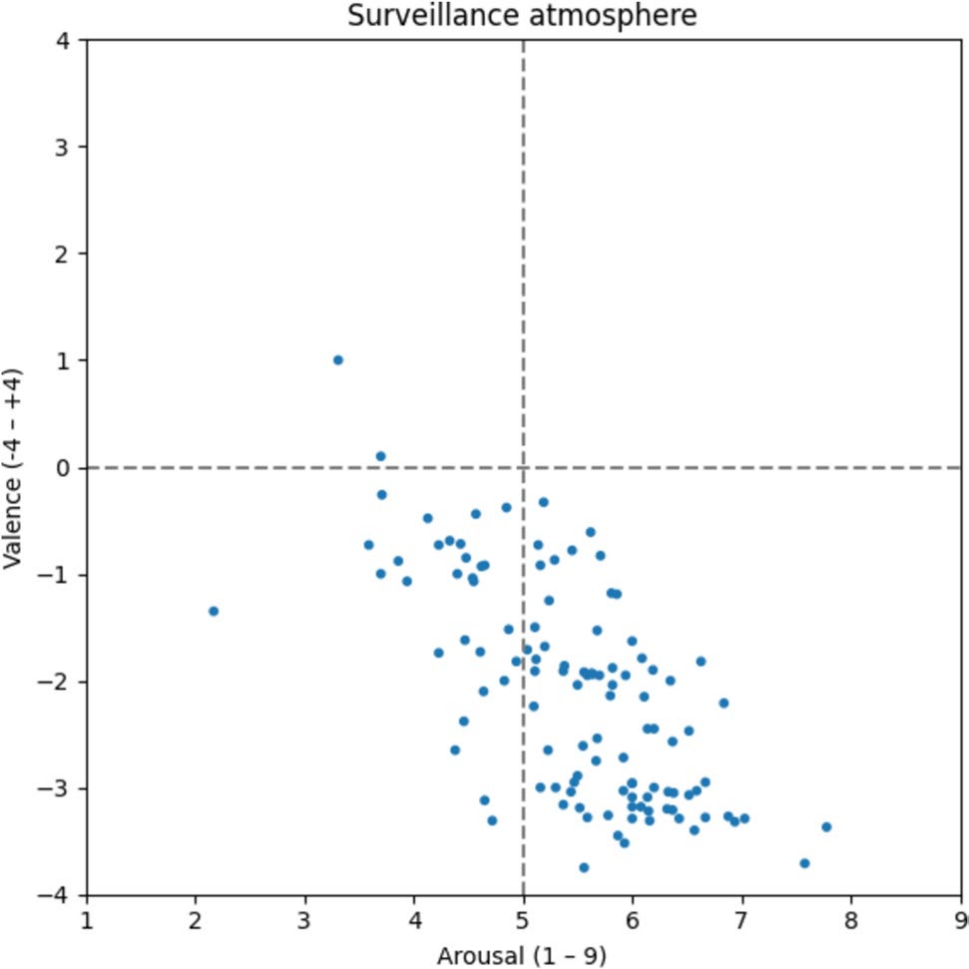
Surveillance atmosphere

With only 114 words, the surveillance atmosphere has received relatively few mentions, distributed similarly to the pressure-laden atmosphere (Fig. 14). Words such as *control* [v: −0.38; a: 4.85; Kontrolle], *suspicious* [v: −2.65; a: 4.38; misstrauisch], *spying* [v: −3.40; a: 6.57; Spionage], *bugging* [v: −3.03; a: 5.92; Abhören], and *video surveillance* [v: −3.22; a: 6.15; Videoüberwachung] are linked to a surveillance atmosphere.

#### Anxiety atmosphere

**Fig. 15:**
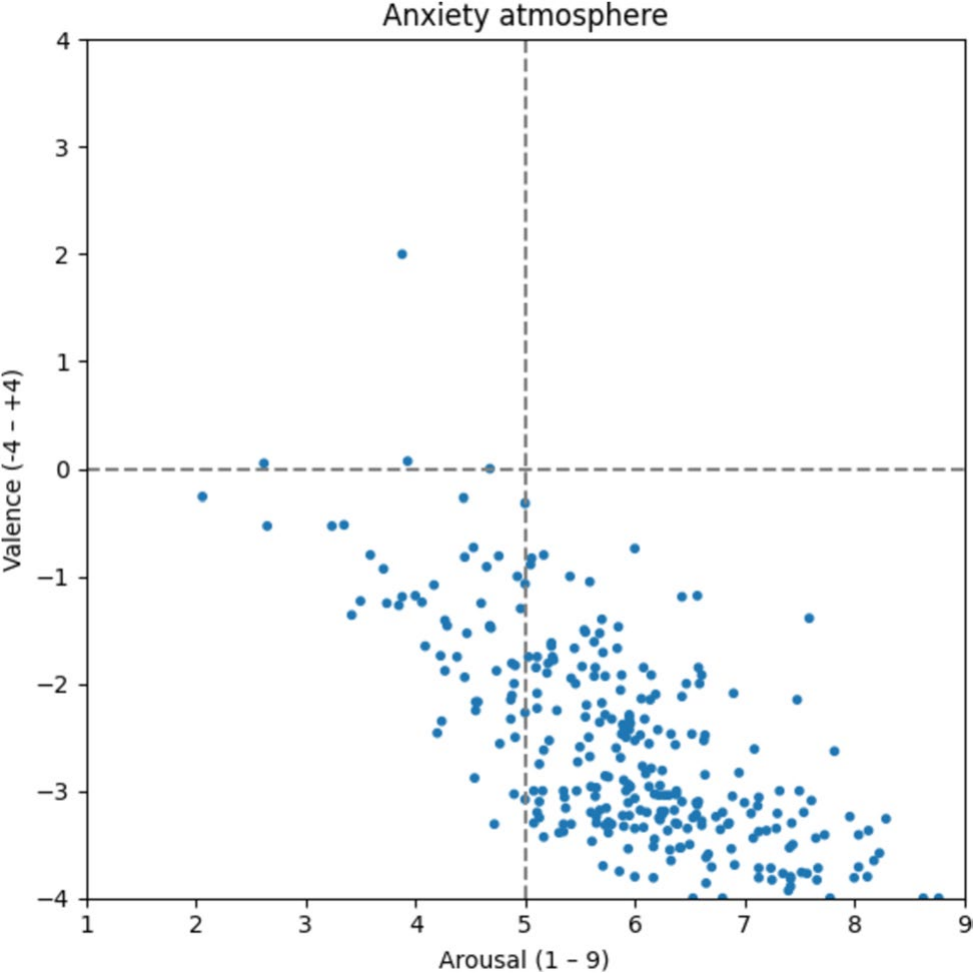
Anxiety atmosphere

The anxiety atmosphere (Fig. 15) stands out with the most negative average ratings (v: −3.56) and the second-highest arousal ratings (a: 5.88), indicating an emotionally charged and potentially distressing atmosphere. Altogether 289 words such as *panic* [v: −3.30; a: 6.37; Panik], to *fear* [v: −3.16; a: 5.74; fürchten], *existential fear* [v: −3.81; a: 6.17; Existenzangst], *danger* [v: −3.32; a: 5.73; Gefahr], *nervous* [v: −1.65; a: 4.09; nervös], and *staff reduction* [v: −2.50, a: 5.92; Personalabbau] are mostly attributed to the lower right quadrant and fit into the picture of an unsettling environment.

#### Toxic atmosphere

**Fig. 16:**
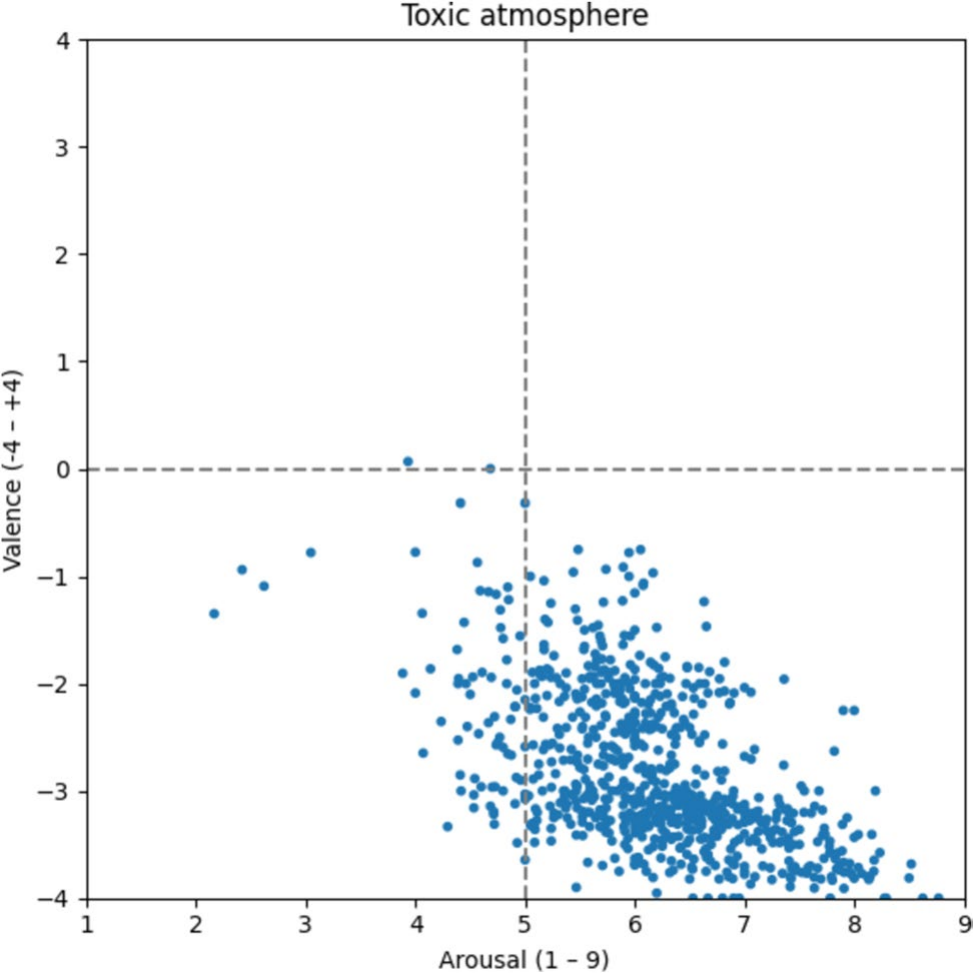
Toxic atmosphere

Finally, 784 words are associated with a toxic atmosphere (Fig. 16). The words are centered in the lower right quadrant as well. On average, they have the highest arousal ratings and the second-highest negative ratings (v: −2.82; a: 6.17), indicating that a toxic atmosphere is very tense and aggravating. The words *evil* [v: −3.32; a: 6.04; böse], *to yell* [v: −3.53; a: 6.41; anschreien], *violence* [v: −3.80; a: 8.12; Gewalt], *poisonous* [v: −3.05; a: 6.75; giftig], and *tyranny* [v: −3.83; a: 8.13; Tyrannei] intuitively fit into the description of a toxic atmosphere.

### Limitations

One of the primary limitations of our study is the length of the survey, which averaged 43 minutes. This extended duration likely contributed to the significant dropout rate, with 34% of participants failing to complete the survey. The repetitive nature of the tasks involved may have induced participant fatigue, potentially compromising the quality of responses due to decreased engagement or attentiveness as the survey progressed. This high attrition rate raises concerns about potential biases in the sample. Participants who completed the survey may systematically differ from those who dropped out, potentially skewing the results. For instance, it is plausible that only the most persistent or highly motivated participants remained until the end, which could lead to a nonrepresentative sample. These differences could affect the generalizability of our findings, as the data may not fully capture the experiences or perceptions of those who disengaged early (Galesic & Bosnjak, 2009).

Nevertheless, these limitations are not unique to GANAiO but are common in similar studies. For instance, the length of our survey aligns with that of Monnier and Syssau (2014), and the number of words evaluated per participant varies significantly across studies. In the studies by Stadthagen-Gonzalez et al. (2017), participants assessed approximately 350 words, in Schmidtke et al. (2014) 603, in Redondo et al. (2007) 1034, and in Moors et al. (2013) even 4300 words. These studies, like ours, faced challenges related to participant fatigue, survey attrition, and potential bias. Therefore, the issues we encountered with survey length and attrition are part of broader methodological concerns in this field.

Second, while the circumplex model of affective atmospheres, as proposed by Julmi (2017a), provides a valuable framework for understanding affective phenomena in organizational environments, it is essential to acknowledge its limitations and that of other circumplex models. Emotions and atmospheres are complex and multifaceted, and reducing them to two dimensions (pleasure–displeasure and arousal–sleepiness or narrowing–widening) might oversimplify their richness and diversity. Therefore, various studies suggest that additional dimensions, such as dominance or control, and contextual factors significantly influence affective experiences (Mehrabian & Russell, 1974; Remmington et al., 2000). As our study focuses only on the dimensions of valence and arousal, environments characterized by high levels of control or stress, for example, may not be fully captured by our dictionary. While the independence of valence and arousal has been established (Russell & Mehrabian, 1977), the dimension of dominance is often overlooked, partly because its independence remains inconclusive (Russell, 1979; Donovan & Rossiter, 1982; Donovan et al., 1994). Furthermore, organizational research on affective atmospheres has not consistently explored the relevance of other dimensions, such as dominance or imageability. Consequently, no widely accepted theory currently justifies the inclusion of additional dimensions beyond valence and arousal when rating affective experiences of atmospheres. Our study further supports this gap, highlighting the need to consider these additional dimensions for a more comprehensive understanding of affective atmospheres in organizations, as demonstrated by the categorical approach. Nevertheless, the categorical approach allows researchers to identify a broader range of factors that are independent of valence and arousal, yet critical to defining specific types of atmospheres. This approach enables a more nuanced understanding of organizational atmospheres by offering a comprehensive set of words that describe different types of affective atmospheres. By incorporating these additional factors, the categorical approach provides a richer and more textured perspective on how affective atmospheres are experienced and understood, addressing the limitations of the dimensional approach.

A further limitation of this study relates to the exploratory nature of the categorization of organizational atmospheres. While we employed a data-driven, inductive approach to identify atmosphere types, the categories remain preliminary due to the current lack of established theoretical frameworks specifically addressing affective atmospheres in organizations. Consequently, certain categories may exhibit overlap, as evidenced by the intersection of terms between categories like “toxic” and “anxiety.” Although some overlap is inherent to the subjective nature of atmospheres, it complicates the distinction between categories, and further refinement is needed to ensure clearer boundaries. Moreover, the exclusion and consolidation of certain categories—such as the merging of “train station” and “call center” atmospheres with the “open-plan office” category—reflects the challenge of differentiating between atmospheres that share similar characteristics or describe spatial contexts rather than distinct emotional atmospheres. While our methodological choices were guided by the data at hand, the exploratory character of these categories may limit the generalizability and precision of the GANAiO dictionary. Future research should aim to validate or reject these categories and explore additional dimensions or characteristics that might further differentiate atmospheres in organizational settings. Given the inductive nature of this approach, we acknowledge that the categories may not yet fully capture the complexity of affective atmospheres across different organizational contexts. As such, this study should be seen as a first step toward building a more comprehensive framework, and further theoretical and empirical work will be necessary to refine and expand upon these findings.

Moreover, our study encountered challenges in the reliable measurement of arousal ratings, which aligns with previous research suggesting that arousal can be particularly difficult to measure consistently (Ekkekakis, 2013). The lack of high activating positive atmospheres in our data does not necessarily imply their absence in real-life organizational settings. Various external factors, such as the nature of work, organizational culture, and individual differences, can affect arousal levels. Therefore, even though our study did not find significant evidence of high activating positive atmospheres, it is plausible that such atmospheres do exist in certain organizational contexts. Future research should employ more robust and diverse methodologies to capture the nuances of arousal in workplace atmospheres.

A further notable limitation of our study pertains to the reliance on user evaluations sourced from the employer review platform *kununu*, which predominantly focuses on German organizations and companies. This geographic and cultural specificity raises concerns regarding the generalizability of our findings beyond the context of German workplaces. Given the potential for cultural bias inherent in the data obtained from a single cultural context, it is essential to acknowledge that the results may not be readily extrapolatable to organizations in other countries or cultures. Cultural nuances, values, and workplace norms vary significantly across different regions, and what constitutes a positive or negative organizational atmosphere can differ markedly between cultures. Therefore, it is crucial to recognize that the findings, based on data from a single cultural context, may not be fully transferable to organizations in other regions or cultures, where workplace norms and perceptions of organizational atmospheres differ.

## Conclusion

We developed GANAiO to advance and contribute to computational research on affective atmospheres in organizations. This involved deriving 5956 words and assigning them to 11 categories representing various types of atmospheres. Following a methodology closely aligned with emotion research studies like ANEW and BAWL-R, the words were rated along the affective dimensions of valence and arousal. According to the notable absence of the typical U curve, we assume that atmospheres and emotions are distinctly different phenomena. Furthermore, the words were rated within the context of the work environment. Arousal may be generally lower in positive atmospheres at work than in other areas, whereas no such difference appears to exist for negative atmospheres.

While valence and arousal are essential dimensions for understanding atmospheres, organizational environments cannot be fully encapsulated by these dimensions alone. To address this limitation, our research examines how various norms are perceived in relation to their capacity to describe organizational atmospheres. This approach highlights the complexity and richness of atmospheres, which go beyond emotional valence and arousal. Additionally, differences within the atmosphere categories revealed distinct qualitative variations despite similar scatter plots, further underscoring the multidimensional nature of organizational atmospheres. All in all, we believe this distinction is crucial for advancing psychological theories related to environmental psychology and organizational behavior.

Based on these considerations and the results of our study, we encourage researchers to replicate our study in diverse linguistic and contextual settings, engaging in a thorough examination of our assumptions. By doing so, a comprehensive and cross-cultural understanding of affective atmospheres in organizations can be achieved. This replication effort will not only contribute to the robustness and generalizability of our findings but also provide an opportunity to uncover potential variations and nuances across different cultural and organizational contexts. Moreover, rigorous testing of our assumptions in varied settings will enhance the external validity of our research, allowing for a more nuanced interpretation of the results. Comparative analyses with findings from diverse cultural backgrounds and organizational environments can uncover universal patterns or context-specific variations in the perception and expression of organizational atmospheres. In addition, our work offers valuable insights and tools that can be utilized in both experimental and applied research settings, thereby benefiting a broad audience within the field of (organizational) psychology.

It must be noted that this study does not offer a qualitative analysis of real atmosphere types. Instead, the use of the dictionary allows researchers to structure large datasets, forming the groundwork for qualitative analyses. By facilitating the identification of factors contributing to typical atmospheres, our approach provides a foundation for developing strategies to foster positive, stimulating environments within organizations. We strongly encourage researchers to leverage the dictionary for laying the groundwork in qualitative analyses and gaining insights into organizational atmospheres.

## Data Availability

All data generated or analyzed during this study are included in this published article.

## Code availability

The analysis scripts are available at OSF (https://osf.io/xqd2c/).
